# Overview and clinical significance of multiple mutations in individual genes in hepatocellular carcinoma

**DOI:** 10.1186/s12885-022-10143-z

**Published:** 2022-10-05

**Authors:** Taisuke Imamura, Yukiyasu Okamura, Keiichi Ohshima, Katsuhiko Uesaka, Teiichi Sugiura, Takaaki Ito, Yusuke Yamamoto, Ryo Ashida, Katsuhisa Ohgi, Shimpei Otsuka, Sumiko Ohnami, Takeshi Nagashima, Keiichi Hatakeyama, Takashi Sugino, Kenichi Urakami, Yasuto Akiyama, Ken Yamaguchi

**Affiliations:** 1 Division of Hepato-Biliary-Pancreatic Surgery, Shizuoka Cancer Center, 1007 Shimonagakubo, Sunto-Nagaizumi, Shizuoka, 4118777 Japan; 2 Department of Digestive Surgery, Nihon University School of Medicine, Tokyo, Japan; 3 Medical Genetics Division, Shizuoka Cancer Center Research Institute, Shizuoka, Japan; 4 Cancer Diagnostics Research Division, Shizuoka Cancer Center Research Institute, Shizuoka, Japan; 5 SRL, Inc., Tokyo, Japan; 6 Division of Pathology, Shizuoka Cancer Center, Shizuoka, Japan; 7 Immunotherapy Division, Shizuoka Cancer Center Research Institute, Shizuoka, Japan; 8 Shizuoka Cancer Center Hospital and Research Institute, Shizuoka, Japan

**Keywords:** Hepatocellular carcinoma, Multiple mutations, Hypermutation, *MUC16*

## Abstract

**Background:**

Multiple mutation (MM) within a single gene has recently been reported as a mechanism involved in carcinogenesis. The present study investigated the clinical significance of MMs in hepatocellular carcinoma (HCC).

**Methods:**

Two hundred twenty-three surgically resected HCCs were subjected to gene expression profiling and whole-exome sequencing.

**Results:**

MMs in individual genes were detected in 178 samples (MM tumors: 79.8%). The remaining samples all carried a single mutation (SM tumors: 20.2%). Recurrence-free survival in the MM group was significantly worse in comparison to the SM group (*P* = 0.012). A Cox proportional hazard analysis revealed that MM tumor was an independent predictor for worse a prognosis (hazard ratio, 1.72; 95% confidence interval, 1.01–3.17; *P* = 0.045). MMs were frequently observed across in various genes, especially MUC16 (15% of samples had at least one mutation in the gene) and CTNNB1 (14%). Although the MUC16 mRNA expression of MUC16 wild-type and MUC16 SM tumors did not differ to a statistically significant extent, the expression in MUC16 MM tumors was significantly enhanced in comparison to MUC16 SM tumors (*P* < 0.001). In MUC16, MMs were associated with viral hepatitis, higher tumor marker levels and vascular invasion. The MUC16 MMs group showed significantly worse recurrence-free survival in comparison to the MUC16 SM group (*P* = 0.022), while no significant difference was observed between the MUC16 SM group and the MUC16 wild-type group (*P* = 0.324).

**Conclusions:**

MM was a relatively common event that may occur selectively in specific oncogenes and is involved in aggressive malignant behavior.

**Supplementary Information:**

The online version contains supplementary material available at 10.1186/s12885-022-10143-z.

## Introduction

Hepatocellular carcinoma (HCC) is the third leading cause of cancer-related death worldwide [1]. Advances in next-generation sequencing have enabled the examination of cancer genomes and led to the discovery of driver alterations. In HCC research, these advances have enabled the processing of the HCC genome, and somatic mutations, structural alterations, HBV integration [2], RNA editing and retrotransposon changes [3] have been identified. Somatic mutation in the *TERT* promoter has been identified as the most frequent alteration (approximately 60%) in HCC [4]. In coding regions, whole-exome sequencing detected frequent mutations in candidate driver genes, such as *TP53* (31%), *ARID1A* (28.2%), *CTNNB1* (18.8%), *MTDH* (14.7%), *AXIN1* (14.2%), *CDKN2A* (11.7%), and *ARID2* (10.9%) [3]. These comprehensive genomic analyses have already identified many pathways as potential therapeutic targets. However, most of the genetic alterations identified were shown to occur with low frequency. Thus, these findings indicate that HCC might be a disease for which the development of molecular targeted treatment is challenging.

Multiple mutations (MMs) in the same oncogene have been newly characterized as one of the mechanisms for the promotion of carcinogenesis [5]. Although tumour suppressor genes (TSGs) are known to be affected by multiple loss-of-function mutations [6, 7], no study has investigated MMs arising in the same oncogene during cancer initiation and development in a structured and consistent way. Saito et al. found that MMs in *PIK3CA*, a representative oncogene, resulted in increased activation of downstream signaling and dependency on the mutated genes as well as an increased sensitivity to specific inhibitors. Furthermore, MMs were reported to be functionally weak, infrequent mutations, preferentially in cis, suggesting that they act in combination to increase oncogenicity [5]. MMs in the same oncogene were reported to be more frequent than expected, with 9% of pan-cancer samples with mutations in oncogenes harboring MMs. These findings indicated that oncogenic MMs are a relatively common driver event, suggesting an underlying mechanism of clonal selection of suboptimal mutations with a low frequency. The previous report was a pan-cancer analysis to overview the landscape of MMs in the same oncogene. Therefore, the frequency and clinical significance of MMs in individual cancer types remain unclear.

Here, we performed comprehensive genetic profiling of HCCs using whole-exome sequencing (WES) and gene expression profiling (GEP) analysis in a large Japanese population. To overview MMs in HCCs and assess the clinical relevance of MMs in HCC patients, we investigated the accumulation of MMs in each gene and the association between MMs and clinicopathological information.

## Methods

### Ethics statement

To investigate the biological characteristics of cancer and diathesis of each patient with cancer, the Shizuoka Cancer Center started Project High-tech Omics-based Patient Evaluation (HOPE) in 2014 [8]. Project HOPE was designed according to the “Ethical Guidelines for Human Genome and Genetic Analysis Research” revised in 2013 [8]. Written consent was obtained from all patients participating in Project HOPE. The present study used the data from Project HOPE and was approved by the Institutional Review Board of Shizuoka Cancer Center (approval no. 25–33). The study protocol conforms to the ethical guidelines of the Declaration of Helsinki.

### Patient selection and study design

From January 2014 to March 2019, 223 surgically resected HCCs were analyzed in Project HOPE. Written informed consent was obtained from all participants. All tumor tissues were pathologically diagnosed as HCC. Tumor tissue samples were dissected from fresh surgical specimens. The surrounding normal tissue was also obtained whenever possible. In addition, peripheral blood was collected as a control for WES. DNA was extracted from tissue samples using a QIAamp DNA Blood MINI Kit (Qiagen, Venlo, The Netherlands). For RNA analysis, tissue samples were submerged in RNAlater solution (Thermo Fisher Scientific), minced and stored at 4 °C overnight before RNA extraction. To validate our findings in other cohort, mutation profiles were extracted from the public database in the TCGA project [9].

### Treatment strategy for HCCs

The details of the surgical strategy and procedure have been previously reported [10]. No preoperative or postoperative adjuvant therapy was performed. Patients underwent physical examinations and blood testing every three months postoperatively. Serial CT or liver ultrasonography was performed in each patient every three to six months. When HCC recurrence was detected, the most appropriate therapy was applied after considering the patient’s liver function and tumor factors. Therapy options included repeat hepatectomy, transcatheter arterial chemoembolization, radiofrequency ablation, or sorafenib.

### WES analysis of HCC tissues using next-generation sequencing

WES analysis was performed as previously described [11, 12]. Briefly, DNA was subjected to WES on an Ion Proton System (Thermo Fisher Scientific). Torrent Suite software (ver. 4.4; Thermo Fisher Scientific) was used to convert binary raw data into sequence reads that were mapped to the reference human genome (UCSC, hg19). The mapping results were stored as BAM files. Two BAM files uploaded to the Ion Reporter system were analyzed simultaneously. For this analysis, AmpliSeq exome tumor-normal pair workflow (ver. 4.4, Thermo Fisher Scientific) with a Custom Hotspot file was used, and this Custom Hotspot file specifies the somatic and pathogenic mutations registered in COSMIC and ClinVar. The sequence data derived from blood samples were used as matched controls. Mutations fulfilling at least one of the following criteria were discarded as false positive: (1) quality score < 60, (2) depth of coverage < 20, (3) variant read observed in one strand only, (4) clipped sequence length < 100 (avg_clipped_length < 100), (5) variant located on either sequence end (avg_pos_as_fraction < 0.05), and (6) mutation matches one on an in-house false-positive list. Parameters specified in criteria (4) and (5) were calculated by bam-readcount with option “-q 1” (ver. 0.8.0) (https://github.com/genome/bam-readcount).

### GEP using DNA microarray analysis

GEP analysis was performed as previously described [11, 13]. Total RNA was extracted using an miRNeasy Mini Kit (Qiagen, Hilden, Germany) according to the manufacturer’s instructions. RNA samples with an RNA integrity number of greater than or equal to 6 were used for DNA microarray analysis. Labeled samples were hybridized to a SurePrint G3 Human Gene Expression 8 × 60 K v2 Microarray (Agilent Technologies, Santa Clara, CA, USA). The fold change between tumor and normal tissues from the same patient was calculated from the normalized values.

### Clinicopathological variables

Data on clinicopathological characteristics were collected from a prospectively maintained HCC database at Shizuoka Cancer Center Hospital. Tumor size was measured at its largest diameter.

### Construction of a catalogue of cancer-related genes

The classification for oncogenes and TSGs were obtained from COSMIC Cancer Gene Census [14], and OncoKB Cancer Gene List [15] as previously described, in our analysis pipeline Shizuoka Multi-omics Analysis Protocol [16].

### CCLE cell line data

The mutation call data (depmap_19Q1_mutation_calls_v2.csv) for 1,601 cell lines and drug-sensitivity data (v17.3_fitted_dose_response.xlsx) for 1,065 cell lines were obtained from the DepMap (https://depmap.org/portal/), CCLE (https://portals.broadinstitute.org/ccle/) and Genomics of Drug Sensitivity in Cancer (GDSC; https://www.cancerrxgene.org/) databases.

### Statistical analyses

Continuous variables were expressed as the median with the interquartile range (IQR) and compared using the Mann–Whitney *U* test or an analysis of variance. AFP and PIVKAII were compared by log-transformation in base 10. A univariate analysis for categorical variables was performed by the chi-square test and Fisher’s exact probability test. For comparisons of three or more groups, *p*-values were adjusted using the Bonferroni method. The thresholds generally accepted in clinical settings were employed as the cut-off value of continuous variables for statistical processing. In the liver damage classification [17], the ICG R_15_ value is classified into three groups by cut-offs at 15% and 40%. Taking this into account, a cut-off value of 20% was used to define the 2 groups in the present study. For AFP and PIVKAII, 200 ng/mL [18] and 100 mAU/mL [19] were used in our study, in reference to previous reports. The overall survival (OS) and relapse-free survival (RFS) were calculated using the Kaplan–Meier method, and the log-rank test was used to evaluate the statistical significance of the differences. A Cox proportional hazard regression analysis was used for the multivariate prognostic analysis. All statistical analyses were performed using the JMP software package, version 14.0 for Mac (SAS Institute Inc., Cary, NC, USA). A *P*-value lower than 0.050 was considered statistically significant.

## Results

### Tumor samples and patients

In total, 223 HCC tissues were analyzed. The median patient age was 71 years old (IQR: 65–77 years). The present cohort consisted of 182 males (82%) and 41 females (18%). Small tumors tended to be excluded from Project HOPE, since the removal of tumor tissue samples in patients with small tumors would make their pathological diagnosis difficult. The study included cases in which resection was performed for recurrence or remnant after previous treatment; 4 cases of transcatheter arterial embolization for rupture (2%), 4 cases of transcatheter arterial chemoembolization (TACE, 2%), 4 cases of raiofrequency ablation (RFA, 2%), 4 cases of RFA with TACE (2%), and 1 case of proton beam treatment (0%) were included. The median tumor size was 35 mm (range: 24–70 mm). The median follow-up period was 34.1 months; the 3-year OS was 81.5% and the median RFS after surgery was 27.6 months.

### Overview of MMs in individual genes

**Fig. 1:**
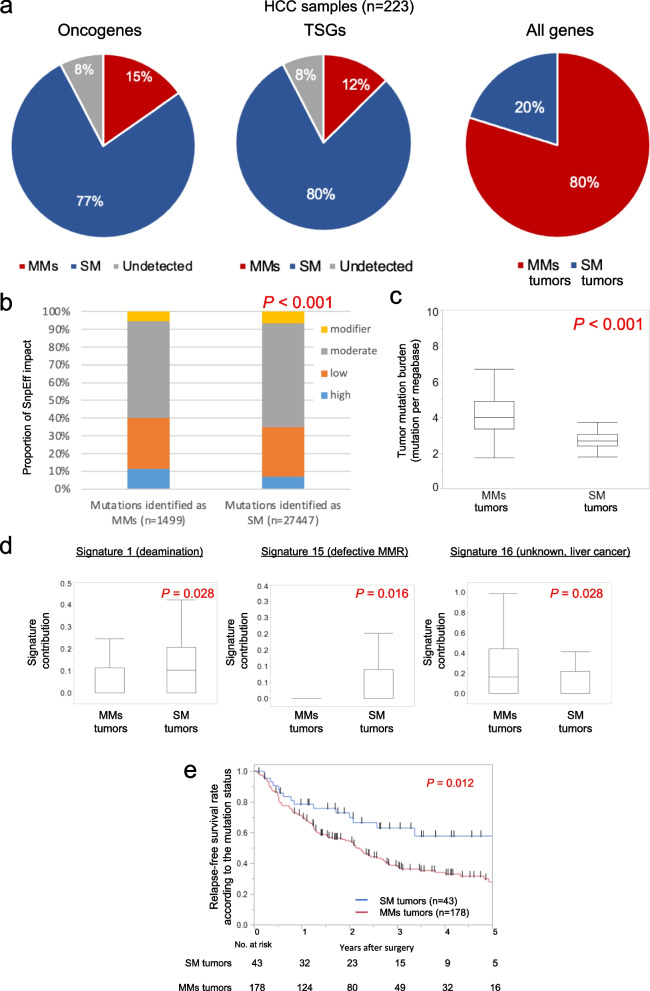
Fraction of multiple mutations in individual genes. **a** Percentages of HCCs with single mutation (SM) and multiple mutations (MMs) according to the classification for oncogenes and tumor suppressor genes (TSGs). **b** Comparison of the impact of genomic variant annotations and functional effect by SnpEff. **c** A comparison of TMB between MMs tumors and SM tumors. The TMB was significantly higher in MMs tumors than that in SM tumors. **d** Comparisons of signature contributions between MMs tumors and SM tumors. Three signature scores were significantly varied betweem MMs tumors and SM tumors. **e** Prognostic analysis according to the presence of MMs. The RFS was significantly worse in the group with MMs tumors than in the group with SM tumors (*P* = 0.012, log-rank test)

In the 223 samples, MMs within a oncogene were identified in 35 (15%) samples and MMs within a TSG were identified in 29 (12%) samples. For all genes, MMs within individual genes was identified in 178 samples (79.8%, MM tumors). All the remaining samples carried single mutation (20.2%, SM tumors, Fig. 1a). To compare the impact of genomic variant annotations and functional effect between mutations identified as SM and mutations identified as MMs, genomic variant were classified by SnpEffs into four levels in accordance with the variety of alterations as follows: high: nonsense mutation, frame-shift mutation and splice site mutation; moderate: missense mutation and in-frame indel; low: synonymous mutation; and modifier: untranslated region mutation. Mutations identified as MMs showed a higher fraction of high impact mutations than mutatios identified as SM; a larger impact on the protein structure caused by amino acid alterations were found in mutations identified as MMs than in mutations identified as SM (Fig. 1b). Furthermore, we evaluated the correlation between tumor mutation burden (TMB) and mutational signatures of the COSMIC database and MMs using deconstructSigs [20]. Supplementary Fig. 1 shows MMs, TMB, and signature contributions in samples with mutation count of > 50. The TMB was significantly higher in MMs tumors than that in SM tumors (Fig. 1c). As shown in Fig. 1d, three signature scores were significantly varied betweem MMs tumors and SM tumors. To assess the clinical impact on the presence of MMs in HCC, we performed prognostic analysis according to the presence of MMs. The RFS was significantly worse in the group with MMs tumors than in the group with SM tumors (*P* = 0.012, Fig. 1e). To consider the potential confounding of TMB with MMs, the prognostic analysis included the TMB. The distribution of TMB is shown in Supplementary Fig. 2. The cut-off value was set to 6.65 as 95% tile. The Cox proportional hazard analysis for RFS of all 223 patients who underwent resection identified MMs as an independent predictor for prognosis (hazard ratio, 1.72; 95% confidence interval, 1.01–3.17; *P* = 0.045) and showed that microvascular invasion (*P* < 0.001) was an independent factor that predicted poor survival (Table 1). No significant prognostic effect was found in the TMB.

**Table 1:** Univariate and multivariate analyses for relapse-free survival after hepatectomy

Variable		Univariate			Multivariate		
		HR	95% CI	*P*-value †	HR	95% CI	*P*-value ‡
Genetic signatures							
Tumor mutation burden							
Hyper	146	1.41	0.97–2.10	0.079			
Hypo	77						
Multiple mutations							
Present	**178**	**1.97**	**1.19–3.53**	**0.012**	**1.72**	**1.01–3.17**	**0.045**
Absent	45						
Clinicopathological factors							
Sex							
Male	182	1.22	0.78–2.01	0.402			
Female	41						
Age, years							
≥ 70	114	0.97	0.69–1.39	0.893			
< 70	109						
HBV or HCV							
Negative	112	0.92	0.65–1.31	0.655			
Positive	111						
ICG-R15, %							
≥ 20	13	1.30	0.64–2.36	0.424			
< 20	210						
AFP, ng/ml							
≥ 200	48	1.40	0.91–2.09	0.106			
< 200	171						
PIVKA-II, mAU/ml							
≥ 100	**121**	**1.84**	**1.27–2.69**	**0.001**	1.34	0.88–2.06	0.177
< 100	95						
Tumor size, mm							
≥ 30	**132**	**1.68**	**1.16–2.46**	**0.006**	1.23	0.84–1.96	0.257
< 30	**91**						
Macrovascular invasion							
Positive	**15**	**2.43**	**1.23–4.32**	**0.004**	1.72	0.86–3.10	0.119
Negative	**208**						
Microvascular invasion							
Positive	**75**	**2.26**	**1.58–3.22**	** < 0.001**	**1.96**	**1.36–2.83**	** < 0.001**
Negative	**148**						
Liver damage							
Liver cirrhosis	88	1.36	0.95–1.94	0.085			
No cirrhosis	135						

Significant values are in bold

*HR* Hazard ratio, *CI* Confidence interval, *HBV* Hepatitis B virus, *HCV* Hepatitis C virus, *ICG* Indocyanine green, *RFS* Relapse-free survival, *AFP* Alpha fetoprotein

^†^Kaplan–Meier method. Significance was determined by the log-rank test

^‡^Multivariate survival analysis was performed using Cox’s proportional hazard model

### Frequent MMs in a variety of oncogenes in HCC

**Fig. 2:**
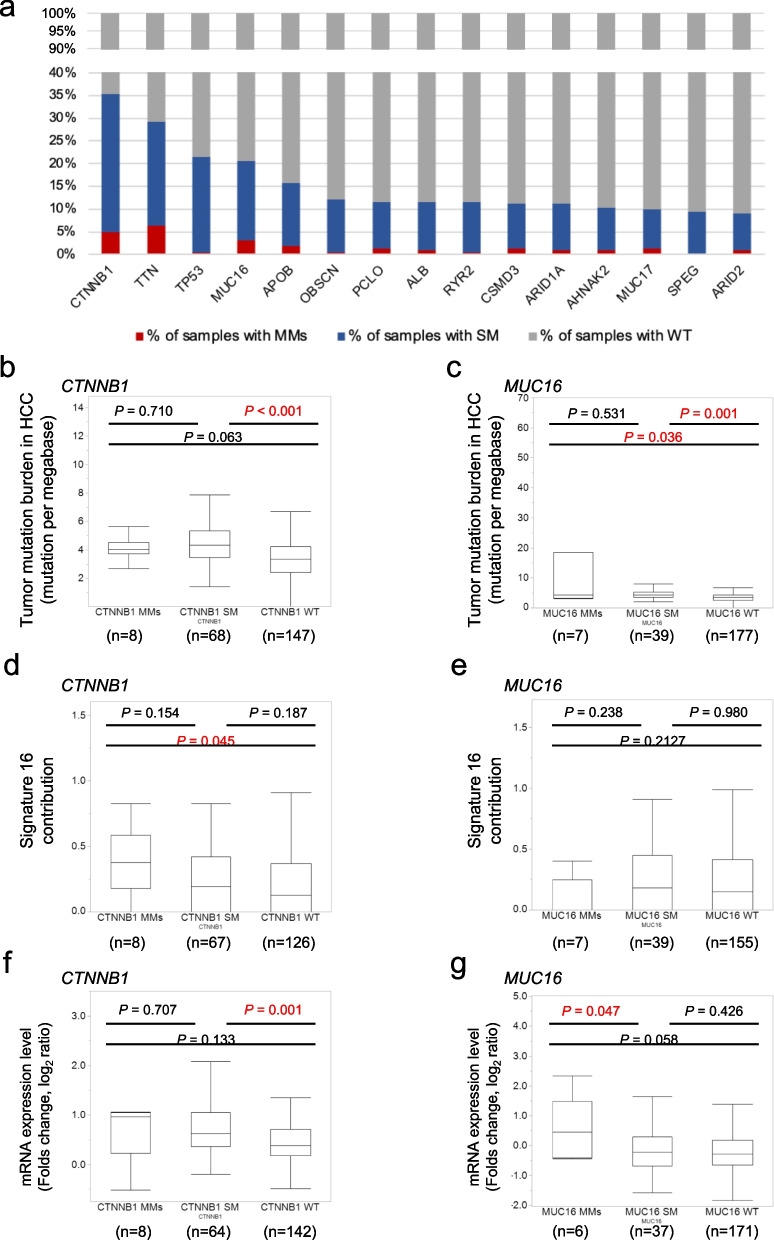
Frequent MMs in a variety of oncogenes in HCC. **a** The percentages of samples with wild type, single mutation, and multiple mutations for 14 genes with 20 or more mutated samples in the present cohort (*n* = 223). Correlations between MMs in *CTNNB1* (**b**) and *MUC16* (**c**) and TMB were found between samples with SM and samples with the wild-type gene (*CTNNB1*, *P* < 0.001; *MUC16*, *P* = 0.001, Mann–Whitney *U* test) but not between SM and MMs (*CTNNB1*, *P* = 0.710, *MUC16*, *P* = 0.531, Mann–Whitney *U* test). The signature 16 was significantly higher in samples with MMs in *CTNNB1* than samples with the wild-type *CTNNB1,* although no significant differences in the signature score between samples with SM in *CTNNB1* and samples with the wild-type *CTNNB1* (**d**)*.* No significant differences in the signature 16 score according tot the mutational status in *MUC16* (**e**). The impact of MMs on gene expression in *CTNNB1* (**f**) and *MUC16* (**g**)

Figure 2a shows the percentages of samples with wild type, single mutations, and multiple mutations for 14 genes with ≥ 20 mutated samples in the present cohort (*n* = 223). MMs were frequently observed across a wide variety of genes; we found that 5% or more of the mutated samples carried MMs across 26 genes, particularly in *MUC16* (15% of samples with mutation in the gene) and *CTNNB1* (14%), suggesting that MMs within individual oncogenes is a relatively common phenomenon. We decided to focus on *CTNNB1* and *MUC16*, which are recognized as being associated with cancer, for a further investigation. To assess the confounding of TMB with MMs, correlations between MMs in *CTNNB1* (Fig. 2b) and *MUC16* (Fig. 2c) and TMB were investigated. In both genes, significant differences in the TMB were found between samples with SM and samples with the wild-type gene (*CTNNB1*, *P* < 0.001; *MUC16*, *P* = 0.001), but no significant differences were found between SM and MMs (*CTNNB1*, *P* = 0.710, *MUC16*, *P* = 0.531). These findings suggest that MMs are not just a reflection of TMB but that there is selection pressure to accumulate mutations in specific genes. Therefore, we evaluated the mutational pattern of MMs in the genes. Using deconstructSigs [20], mutational signatures of the COSMIC database were investigated. The liver-cancer-specific signature 16 [21] was significantly higher in samples with MMs in *CTNNB1* than samples with the wild-type *CTNNB1,* although no significant differences in the signature score between samples with SM in *CTNNB1* and samples with the wild-type *CTNNB1* (Fig. 2d)*.* No significant differences in the signature 16 score between samples with MMs in *MUC16* and samples with the wild-type *MUC16* and between samples with SM in *MUC16* and samples with the wild-type *MUC16* were confirmed (Fig. 2e). The details of all mutations identified in *MUC16*, including protein change and DNA change are presented in Supplementary Table 1. The distribution of mutations and fraction of MMs for each position in *CTNNB1* and *MUC16* are shown in Supplementary Fig. 3. In *CTNNB1*, most mutations were located in major hotspots of exon 3. No significant difference of the frequency was observed between *CTNNB1* SM tumors and *CTNNB1* MMs tumors. In *MUC16*, mutations frequently located at exon 3 and there was no significant difference in the frequency between *MUC16* SM and *MUC16* MMs tumors. We investigated the allelic configuration of MMs by phasing from WES reads, which revealed that most MMs (83%) in *CTNNB1* were present in cis. While all of the MMs in *MUC16* was not located in a same amplicon, therefore the allelic configuration of MMs in *MUC16* could not be investigated in the present study. Next, we investigated the impact of MMs on gene expression in *CTNNB1* (Fig. 2f) and *MUC16* (Fig. 2g). The gene expression raw data, including expression in tumors, expression in non-tumor areas as normal tissue, and fold-changes, in all cases are shown in Supplementary Table 2. In *MUC16*, MMs had larger alterations of gene expression; although there was no significant difference of expression between samples with wild-type *MUC16* and samples with SM in *MUC16*, the expression in samples with MMs in *MUC16* was significantly enhanced compared with samples with SM in *MUC16* (*P* = 0.047). These results suggest that individually suboptimal mutations can confer enhanced oncogenic potential in combination as MMs. Based on the findings, for further investigation, we focused on *MUC16* as a candidate oncogene to validate the impact of MMs.

### Functional relevance of MMs in oncogenes

To assess the impact of MMs on phenotypes in cancer cell lines, an analysis of drug sensitivity screens in Cancer Cell Line Encyclopedia (CCLE) cell lines [22] was performed. Box plots (Supplementary Fig. 4) show sensitivity to regorafenib for 27 CCLE liver cancer cell lines, according to *MUC16* mutational status. The results revealed that cells harboring in MMs in *MUC16* exhibited a tendency of higher sensitivity to regorafenib than those with no or single *MUC16* mutations, pointing to the potential value of MMs as a predictive marker for targeted therapies.

### Clinical outcomes and MMs in MUC16

**Fig. 3:**
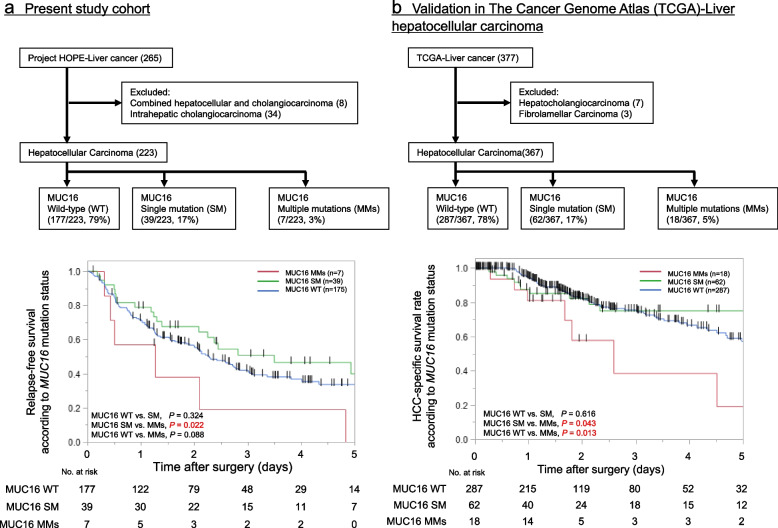
Prognostic impact of MMs in *MUC16* and its validation in TCGA. The frequency of SM and MMs in MUC16 was 17% and 3% in our study and 17% and 5% in TCGA. **a** Patient RFS was significantly worse in the group with *MUC16* MMs than in the group with *MUC16* SM (*P* = 0.022), although there was no significant difference between the group with *MUC16* SM and the group with wild-type *MUC16* in our cohort (*P* = 0.324, log-rank test). **b** In TCGA data sets, no significant differences in HCC-specific survival were observed between the group with *MUC16* SM and the group with wild-type *MUC16* (*P* = 0.616). Patient HCC-specific survival was significantly worse in the group with *MUC16* MMs than in the group with *MUC16* SM (*P* = 0.043) and in the group with wild-type *MUC16* (*P* = 0.013)

To assess the clinical impact of MMs in individual oncogenes, the clinicopathological factors according to mutational status in *MUC16* were investigated (Table 2). MMs in *MUC16* were associated with hepatitis C (*P* < 0.001), increased PIVKAII levels (*P* = 0.032) and macro vascular invasion (*P* = 0.041). Patient RFS was significantly worse in the group with *MUC16* MMs than in the group with *MUC16* SM (*P* = 0.022), although there was no significant difference between the group with *MUC16* SM and the group with wild-type *MUC16* (*P* = 0.324, Fig. 3a). Using TCGA data sets, we checked HCC-specific survival according to mutational status in *MUC16* (Fig. 3b). No significant differences in Kaplan–Meier survival curves were observed between the group with *MUC16* SM and the group with wild-type *MUC16* (*P* = 0.616). Patient HCC-specific survival was significantly worse in the group with *MUC16* MMs than in the group with *MUC16* SM (*P* = 0.043) and in the group with wild-type *MUC16* (*P* = 0.013).

**Table 2:** Clinicopathological factors according to the number of *MUC16* mutations

Variable	Wild-type	Single mutation	Multiple mutations	*P*-value
	*N* = 177	*N* = 39	*N* = 7	
Gender, N, (%)				
Male	145 (82%)	31 (79%)	6 (86%)	** < 0.001**
Female	32 (18%)	8 (21%)	1 (14%)	
Age, years, (IQR)	70 (64–76)	74 (68–78)	65 (64–73)	**0.022**
HBV or HCV, N, (%)				
HCV	53 (30%)	11 (28%)	5 (71%)	** < 0.001**
HBV	34 (19%)	8 (21%)	0 (0%)	
NBNC	90 (51%)	20 (51%)	2 (29%)	
ICG-R15, %, (IQR)	9.5 (6.1–13.7)	10.7 (7.5–15.7)	13.4 (5.9–15.1)	0.445
AFP, ng/ml, (IQR)	9.2 (3.7–144.1)	5.2 (2.3–24.2)	641.4 (7.4–883.7)	0.107
PIVKAII, mAU/ml, (IQR)	116.0 (32.6–1495.0)	414.0 (24.8–3537.5)	5670.0 (160.0–37,800.0)	**0.032**
Tumor size, mm, (IQR)	35 (22–65)	36 (25–76)	63 (33–78)	
Macrovascular invasion, N, (%)				
Positive	12 (7%)	1 (3%)	2 (29%)	**0.041**
Microvascular invasion, N, (%)				
Positive	58 (33%)	15 (38%)	2 (29%)	0.761

Continuous variables expressed as median and interquartile range (IQR)

Significant values are in bold

*HBV* Hepatitis B virus, *HCV* Hepatitis C virus, *ICG* Indocyanine green, *AFP* Alpha fetoprotein, *NBNC* Non B non C

## Discussion

In this study, we performed comprehensive WES for 223 HCCs. The results revealed that a high frequency of mutations in genes did not always correspond to a high frequency of MMs. Instead, MMs were revealed to accumulate selectively in specific genes. The signature of mutations identified as MMs, including mutation type and functional impact, also varied compared with that of mutations identified as SM. Furthermore, the GEP results implied that MMs had a greater impact on gene expression than SMs in some oncogenes, a trend was identified that MMs led to additional up-regulation (gain-of-function) of gene expression. Consequently, MMs are not just a reflection of mutation burden but occur in specific genes and pathways and thus contribute to carcinogenesis and/or acquisition of malignant potential in HCC.

The overall landscape of MMs was recently reported through a pan-cancer analysis of 60,954 cancer samples [5]. The study identified that oncogenic MMs were a relatively common driver event and thus MMs provide a novel underlying mechanism for cancer development. These observations reinforce the idea that MMs in the same oncogene cooperate to potentiate tumor-promoting activity. These findings also indicate the potential usefulness of MMs as a biomarker and a target for molecular-targeted therapy. However, the signature and clinical relevance of MMs in HCCs had remained unclear. In the present study, detailed clinical information led to the validation of the clinical significance of MMs in HCCs, and the present results identified MMs as an independent predictor for prognosis in HCC. To date, there has been no report on the clinical relevance of MMs, and therefore the present study provides further evidence of the prognostic impact of MMs.

The clinical impact of MMs in some genes in a sample led us to assess the clinical impact of MMs in individual oncogenes. Aberrant activation of WNT/β-catenin signaling is a driving molecular event in a wide range of tumors, including HCCs [23]. Somatic missense mutations in exon 3 of *CTNNB1* are frequently reported in HCCs (10.0%–32.8% in genome-wide sequencing studies) [3, 24]. Consistent with previous reports, we found that mutations in *CTNNB1* were frequently identified in HCC and MMs in *CTNNB1* were also frequently found in 14% (11/79) of samples with at least one mutation. However, a significant prognostic difference between SM and MMs in *CTNNB1* was not identified (data not shown). The conflicting prognostic impact of mutated *CTNNB1* due to the bilateral nature was reported. HCC cases with the existence of an interaction between WNT activation and TGF-beta activation show poor survival, whereas HCCs harboring mutant *CTNNB1* show generally favorable prognosis [25]. Therefore, for further investigation, we focused on *MUC16*, in which MMs were identified in 15.2% (7/46) of mutated samples, as a candidate oncogene to validate the impact of MMs.

*MUC16* encodes a protein also known as ovarian carcinoma antigen CA125, which is clinically recommended as a screening biomarker for ovarian cancer. Although CA125 is not a general biomarker for HCC, high preoperative serum CA125 levels have been reported to serve as an independent prognostic factor for the OS and RFS in HCC [26]. MUC16 is an important membrane protein that maintains the normal cellular function and is involved in cancer development. Knockdown of MUC16 in HCC cell lines revealed that MUC16 plays a suppressive role in migration and invasion [26]. Furthermore, it has been reported that MUC16 promotes proliferation and invasion via activation of the JAK2/STAT3 pathway in cervical cancer [27]. Thus, alterations in MUC16 enhance the tumor invasive potential. In contrast, an increase in CA125 in serum was associated with an increase in malignancy and mortality, suggesting that mutations in MUC16 may affect the structural stability of MUC16, causing the tumor-derived protein to shed from the surface of hepatocarcinoma cells. Although the differences between SMs and MMs require further investigation, this may explain the association between mutations in *MUC16* and high tumor marker levels and vascular invasion. The present results demonstrated that the presence of MMs in *MUC16* was associated with higher tumor markers and vascular invasion. Patient RFS was significantly worse in the group with MMs than in the group with SM*.* The findings support the idea that MMs in the same oncogene cooperate to potentiate tumor-promoting activity.

The findings that the accumulation of MMs in specific oncogenes led us to assess the impact of MMs on phenotypes in cancer cell lines. Analysis of drug sensitivity screens in CCLE cell lines [22] revealed that cells harboring in MMs in *MUC16* exhibited a higher sensitivity to regorafenib than those with no or single *MUC16* mutations, indicating the potential value of MMs as a predictive marker for targeted therapies. Similarly, a previous study [5] reported that cells harboring MMs in *PIK3CA* exhibited a higher sensitivity to PI3K inhibitors compared with those with no or single *PIK3CA* mutations, suggesting that MMs may be useful as predictive markers for targeted therapies. The correlation between mutational status in an oncogene and the sensitivity to the targeted drug for the molecule should be further explored in future studies.

During recent years, new immune-modulatory agents have been introduced for HCC treatment, eventually leading to the clinical breakthrough of immune checkpoint inhibitors (ICIs) targeting programmed death-1 (PD-1), programmed death-ligand 1 (PD-L1), or cytotoxic T lymphocyte antigen-4 (CTLA-4) [28]. The TMB, which correlates with MMs, has received increasing attention owing to its potential to estimate the efficacy of the response to ICIs. The present results indicated that MMs were associated with the TMB to some extent; however, MMs were not just a reflection of mutation burden but occurred in specific genes and pathways through a selection mechanism to contribute to carcinogenesis and/or acquisition of malignant potential. These findings support a potential association between MMs and sensitivity to ICIs. Although we cannot draw any conclusions from these results, it may be worthwhile to examine MMs and the sensitivity to ICIs for future perspectives.

This study had several limitations. First, the number of MMs in each specific gene was relatively small, and therefore investigation of the clinical significance of MMs in each gene could not be conducted because of under power statistics. Ideally, the clinical significance of MMs in specific candidate genes will be investigated to establish a biomarker for targeted therapy. Second, the previous study [5] reported that the proportion of MMs in cis was particularly high (86%) in oncogenes with MMs. Consistent with the previous reports, most MMs (83%) in *CTNNB1* were present in cis in the present study. However, all of the MMs in *MUC16* was not located in a same amplicon, therefore the allelic configuration of MMs in *MUC16* could not be investigated.

## Conclusions

MMs are a relatively common event that selectively occurs in specific oncogenes and is involved in aggressive malignant behavior. This is the study investigating the clinical significance of MMs in patients with HCC and provide important insights into the development of personalized treatment strategies for HCCs.

## Supplementary Information

**Additional file 1: Supplementary Fig. 1.** Mutation signature in the sample with multiple mutations. **Supplementary Fig. 2.** The distribution of TMB in HCCs. **Supplementary Fig. 3.** The mutational mapping of MMs in *CTNNB1* and *MUC16. ***Supplementary Fig. 4.** Drug sensitivity screens in Cancer Cell Line Encyclopedia.

**Additional file 2: Supplementary Table 1.** Mutations identified in MUC16.

**Additional file 3: Supplementary Table 2.** Gene expression raw data.

## Data Availability

Data for this study is confidential patient information regulated by the IRB of the institution. The datasets generated and/or analyzed during the current study are available in the National Bioscience Database Center repository (accession no. hum0127; https://humandbs.biosciencedbc.jp/en/).

## Abbreviations

CCLE: Cancer Cell Line Encyclopedia
CTLA-4: Cytotoxic T lymphocyte antigen‐4
GEP: Gene expression profiling
HOPE: High-tech Omics-based Patient Evaluation
ICIs: Immune checkpoint inhibitors
MMs: Multiple mutations
PD-1: Programmed death-1
PD-L1: Programmed death-ligand 1
OS: Overall survival
RFS: Relapse-free survival
WES: Whole-exome sequencing
