# Supplementary material for: Overview and clinical significance of multiple mutations in individual genes in hepatocellular carcinoma
Source: BMC Cancer. 2022 Oct 5;22:1046. doi: 10.1186/s12885-022-10143-z (PMC9535898; doi:10.1186/s12885-022-10143-z)
Supplement: Supplementary file 1 — Additional file 1: Supplementary Fig. 1. Mutation signature in the sample with multiple mutations. Supplementary Fig. 2. The distribution of TMB in HCCs. Supplementary Fig. 3. The mutational mapping of MMs in CTNNB1 and MUC16. Supplementary Fig. 4. Drug sensitivity screens in Cancer Cell Line Encyclopedia. [file 12885_2022_10143_MOESM1_ESM.docx]

**Supplementary Fig. 1** **Mutation signature in the sample with multiple mutations.** Multiple mutations, tumor mutation burden (TMB) and signature contributions are shown in the top, middle and bottom panels, respectively. Each row represents a sample. Signatures in a sample with mutation count of >50 are shown. Proposed etiologies described in COSMIC are shown in parentheses. Signatures with no information regarding etiology are labeled as unknown

**Supplementary Fig. 2** **The distribution of TMB in HCCs.**

The cutoff value was set to 6.65 as 95% tile.

**Supplementary Fig. 3** **The mutational mapping of MMs in *CTNNB1* and *MUC16.***

In *CTNNB1*, most mutations were located in major hotspots of exon 3. No significant difference of the frequency was observed between *CTNNB1* SM tumors and *CTNNB1* MMs tumors. In *MUC16*, mutations frequently located at exon 3 and there was no significant difference in the frequency between *MUC16* SM and *MUC16* MMs tumors.

**Supplementary Fig. 4** **Drug sensitivity screens in Cancer Cell Line Encyclopedia**

To assess the impact of MMs on phenotypes in cancer cell lines, an analysis of drug sensitivity screens in Cancer Cell Line Encyclopedia (CCLE) cell lines ^10^ was performed. Box plots show sensitivity to regorafenib for 27 CCLE liver cancer cell lines, according to mutational status. Cells harboring *MUC16* MMs exhibited higher sensitivity to regorafenib than those with no or single *MUC16* mutations, pointing to the potential value of MMs as a predictive marker for targeted therapies.
