# Supplementary material for: Overview and clinical significance of multiple mutations in individual genes in hepatocellular carcinoma
Source: BMC Cancer. 2022 Oct 5;22:1046. doi: 10.1186/s12885-022-10143-z (PMC9535898; doi:10.1186/s12885-022-10143-z)
Supplement: Supplementary file 2 — Additional file 2: Supplementary Table 1. Mutations identified in MUC16. [file 12885_2022_10143_MOESM2_ESM.docx]

| **Supplementary Table 1.** Mutations identified in MUC16 | | | | | | | | | | | | | | | |
| --- | --- | --- | --- | --- | --- | --- | --- | --- | --- | --- | --- | --- | --- | --- | --- |
|  |  | Mutation 1 | | | |  | Mutation 2 | | | |  | Mutation 3 | | | |
| **Sample** |  | **Protein change** | **Coding DNA change** | **SnpEff impact** | **SnpEff effect** |  | **Protein change** | **Coding DNA change** | **SnpEff impact** | **SnpEff effect** |  | **Protein change** | **Coding DNA change** | **SnpEff impact** | **SnpEff effect** |
| MMs-1 |  | Lys9606* | c.28816A>T | high | stop_gained |  | Pro9270Arg | c.27809C>G | moderate | missense |  |  |  |  |  |
| MMs-2 |  | Pro6502Gln | c.19505C>A | moderate | missense |  | Ser8714Ser | c.26142T>C | low | synonymous |  |  |  |  |  |
| MMs-3 |  | His11763Asn | c.35287C>A | moderate | missense |  | Leu5485Arg | c.16454T>G | moderate | missense |  |  |  |  |  |
| MMs-4 |  | Thr9383Ile | c.28148C>T | moderate | missense |  | Ala1413Thr | c.4237G>A | moderate | missense |  | Gly1060Arg | c.3178G>A | moderate | missense |
| MMs-5 |  | Arg12878Lys | c.38633G>A | moderate | missense |  | Ser8213Asn | c.24638G>A | moderate | missense |  |  |  |  |  |
| MMs-6 |  | Ala7507Thr | c.22519G>A | moderate | missense |  | Thr945Met | c.2834C>T | moderate | missense |  |  |  |  |  |
| MMs-7 |  | Asp12182Gly | c.36545A>G | moderate | missense |  | Thr3883Thr | c.11649T>C | low | synonymous |  |  |  |  |  |
| SM-1 |  | Thr1708Asn | c.5123C>A | moderate | missense |  |  |  |  |  |  |  |  |  |  |
| SM-2 |  | Val4848Val | c.14544C>G | low | synonymous |  |  |  |  |  |  |  |  |  |  |
| SM-3 |  | Val12522Val | c.37566G>A | low | synonymous |  |  |  |  |  |  |  |  |  |  |
| SM-4 |  | Pro6702Leu | c.20105C>T | moderate | missense |  |  |  |  |  |  |  |  |  |  |
| SM-5 |  | Val4305Leu | c.12913G>C | moderate | missense |  |  |  |  |  |  |  |  |  |  |
| SM-6 |  | Gly3756Val | c.11267G>T | moderate | missense |  |  |  |  |  |  |  |  |  |  |
| SM-7 |  | Pro8617Thr | c.25849C>A | moderate | missense |  |  |  |  |  |  |  |  |  |  |
| SM-8 |  | Leu12967His | c.38900T>A | moderate | missense |  |  |  |  |  |  |  |  |  |  |
| SM-9 |  | Ser3288Thr | c.9862T>A | moderate | missense |  |  |  |  |  |  |  |  |  |  |
| SM-10 |  | Pro670Gln | c.2009C>A | moderate | missense |  |  |  |  |  |  |  |  |  |  |
| SM-11 |  | Thr4833Ile | c.14498C>T | moderate | missense |  |  |  |  |  |  |  |  |  |  |
| SM-12 |  | Thr12202Ala | c.36604A>G | moderate | missense&splice_region | | |  |  |  |  |  |  |  |  |
| SM-13 |  | Val10825Phe | c.32473G>T | moderate | missense |  |  |  |  |  |  |  |  |  |  |
| SM-14 |  | Thr11007Ala | c.33019A>G | moderate | missense |  |  |  |  |  |  |  |  |  |  |
| SM-15 |  | Asp735Gly | c.2204A>G | moderate | missense |  |  |  |  |  |  |  |  |  |  |
| SM-16 |  | Pro2220Thr | c.6658C>A | moderate | missense |  |  |  |  |  |  |  |  |  |  |
| SM-17 |  | Val151Ala | c.452T>C | moderate | missense |  |  |  |  |  |  |  |  |  |  |
| SM-18 |  | Arg3573Cys | c.10717C>T | moderate | missense |  |  |  |  |  |  |  |  |  |  |
| SM-19 |  | Val343Ile | c.1027G>A | moderate | missense |  |  |  |  |  |  |  |  |  |  |
| SM-20 |  | Gly12525Ala | c.37574G>C | moderate | missense |  |  |  |  |  |  |  |  |  |  |
| SM-21 |  | Trp3999* | c.11997G>A | high | stop_gained |  |  |  |  |  |  |  |  |  |  |
| SM-22 |  | Pro819Leu | c.2456C>T | moderate | missense |  |  |  |  |  |  |  |  |  |  |
| SM-23 |  | His12349Arg | c.37046A>G | moderate | missense |  |  |  |  |  |  |  |  |  |  |
| SM-24 |  |  | c.*13G>A | modifier | 3_prime_UTR |  |  |  |  |  |  |  |  |  |  |
| SM-25 |  | Leu14196Leu | c.42586T>C | low | synonymous |  |  |  |  |  |  |  |  |  |  |
| SM-26 |  | Gln14374Arg | c.43121A>G | moderate | missense |  |  |  |  |  |  |  |  |  |  |
| SM-27 |  | Glu1433Lys | c.4297G>A | moderate | missense |  |  |  |  |  |  |  |  |  |  |
| SM-28 |  | Met9683Lys | c.29048T>A | moderate | missense |  |  |  |  |  |  |  |  |  |  |
| SM-29 |  | Asp3596Gly | c.10787A>G | moderate | missense |  |  |  |  |  |  |  |  |  |  |
| SM-30 |  | Thr11443Arg | c.34328C>G | moderate | missense |  |  |  |  |  |  |  |  |  |  |
| SM-31 |  | Leu4471Ser | c.13412T>C | moderate | missense |  |  |  |  |  |  |  |  |  |  |
| SM-32 |  | Pro11628Leu | c.34883C>T | moderate | missense |  |  |  |  |  |  |  |  |  |  |
| SM-33 |  | Pro835fs | c.2500_2501delAG | high | frameshift |  |  |  |  |  |  |  |  |  |  |
| SM-34 |  | Thr8863Ser | c.26588C>G | moderate | missense |  |  |  |  |  |  |  |  |  |  |
| SM-35 |  | Val8883Val | c.26649G>T | low | synonymous |  |  |  |  |  |  |  |  |  |  |
| SM-36 |  | Gly13626Gly | c.40878T>C | low | synonymous |  |  |  |  |  |  |  |  |  |  |
| SM-37 |  | Gly8683Glu | c.26048G>A | moderate | missense |  |  |  |  |  |  |  |  |  |  |
| SM-38 |  | Pro7678Pro | c.23034T>C | low | synonymous |  |  |  |  |  |  |  |  |  |  |
| SM-39 |  | Leu3601* | c.10802T>A | high | stop_gained |  |  |  |  |  |  |  |  |  |  |
