# Supplementary material for: Overview and clinical significance of multiple mutations in individual genes in hepatocellular carcinoma
Source: BMC Cancer. 2022 Oct 5;22:1046. doi: 10.1186/s12885-022-10143-z (PMC9535898; doi:10.1186/s12885-022-10143-z)
Supplement: Supplementary file 3 — Additional file 3: Supplementary Table 2. Gene expression raw data. [file 12885_2022_10143_MOESM3_ESM.docx]

| Supplementary Table 2. Gene expression raw data | | | | | | | | | |
| --- | --- | --- | --- | --- | --- | --- | --- | --- | --- |
|  | MUC16 | | | |  | CTNNB1 | | | |
| Sample | Mutation | Tumor | Normal | Fold change (log2 ratio) |  | Mutation | Tumor | Normal | Fold change (log2 ratio) |
| 1 | MMs | -4.53 | -5.73 | 1.20 |  | SM | 1.51 | 0.07 | 1.44 |
| 2 | MMs | -4.86 | -5.13 | 0.27 |  | WT | 0.56 | -0.45 | 1.01 |
| 3 | MMs | -3.15 | -5.49 | 2.34 |  | WT | 0.32 | -0.24 | 0.56 |
| 4 | MMs | -6.01 | -6.66 | 0.65 |  | WT | 1.02 | 0.18 | 0.84 |
| 5 | MMs | -6.82 | -6.42 | -0.40 |  | WT | 0.13 | 0.60 | -0.47 |
| 6 | MMs | -7.10 | -6.66 | -0.44 |  | WT | 0.70 | 0.51 | 0.19 |
| 7 | MMs | -4.98 | NA | NA |  | WT | 0.30 | NA | NA |
| 8 | SM | -5.96 | -6.05 | 0.09 |  | SM | 0.71 | 0.09 | 0.63 |
| 9 | SM | -5.87 | -5.15 | -0.72 |  | WT | 0.25 | -0.17 | 0.42 |
| 10 | SM | -5.84 | -5.96 | 0.12 |  | MMs | 0.40 | 0.07 | 0.33 |
| 11 | SM | -5.69 | -5.78 | 0.09 |  | WT | 0.92 | 0.47 | 0.44 |
| 12 | SM | -6.15 | -5.97 | -0.17 |  | WT | 0.14 | 0.19 | -0.06 |
| 13 | SM | -5.81 | -6.88 | 1.07 |  | SM | 0.90 | 0.18 | 0.72 |
| 14 | SM | -5.89 | -6.19 | 0.30 |  | SM | 1.88 | 0.16 | 1.72 |
| 15 | SM | -3.52 | -4.83 | 1.31 |  | WT | 0.71 | 0.23 | 0.48 |
| 16 | SM | -5.31 | -6.11 | 0.80 |  | WT | 0.42 | 0.30 | 0.12 |
| 17 | SM | -6.66 | -6.33 | -0.33 |  | WT | 0.69 | 0.42 | 0.26 |
| 18 | SM | -5.61 | -5.93 | 0.32 |  | SM | 0.95 | 0.54 | 0.40 |
| 19 | SM | -5.96 | -5.25 | -0.71 |  | WT | 1.44 | 0.41 | 1.03 |
| 20 | SM | -5.94 | -5.29 | -0.65 |  | MMs | 1.29 | 0.32 | 0.97 |
| 21 | SM | -5.63 | -6.19 | 0.56 |  | WT | 0.57 | 0.54 | 0.03 |
| 22 | SM | -6.45 | -5.83 | -0.62 |  | WT | 0.67 | 0.31 | 0.35 |
| 23 | SM | -6.66 | -5.68 | -0.99 |  | SM | 0.75 | 0.08 | 0.67 |
| 24 | SM | -5.44 | -5.22 | -0.22 |  | WT | 0.48 | -0.39 | 0.87 |
| 25 | SM | -5.42 | -4.34 | -1.08 |  | SM | 0.57 | -1.09 | 1.67 |
| 26 | SM | -6.47 | -6.42 | -0.06 |  | SM | 1.30 | 0.23 | 1.06 |
| 27 | SM | -6.01 | -5.64 | -0.37 |  | WT | 1.01 | -0.28 | 1.29 |
| 28 | SM | -6.44 | -5.92 | -0.52 |  | WT | 1.29 | 0.52 | 0.77 |
| 29 | SM | -6.59 | -5.86 | -0.73 |  | WT | 0.52 | 0.30 | 0.22 |
| 30 | SM | -5.50 | -5.67 | 0.17 |  | MMs | 1.63 | 0.67 | 0.96 |
| 31 | SM | -6.00 | -5.55 | -0.45 |  | WT | 0.57 | 0.19 | 0.38 |
| 32 | SM | -5.92 | NA | NA |  | WT | 0.17 | NA | NA |
| 33 | SM | -3.96 | -4.62 | 0.66 |  | WT | 0.84 | 0.14 | 0.70 |
| 34 | SM | -6.04 | -5.29 | -0.74 |  | SM | 0.35 | -0.02 | 0.37 |
| 35 | SM | -6.67 | -5.78 | -0.89 |  | SM | 0.25 | 0.22 | 0.03 |
| 36 | SM | -6.53 | -5.42 | -1.12 |  | SM | 0.59 | 0.28 | 0.31 |
| 37 | SM | -6.01 | NA | NA |  | SM | 0.12 | NA | NA |
| 38 | SM | -6.34 | -6.19 | -0.15 |  | SM | 0.40 | 0.03 | 0.38 |
| 39 | SM | -6.27 | -6.04 | -0.23 |  | WT | 0.28 | 0.28 | 0.00 |
| 40 | SM | -6.24 | -5.80 | -0.44 |  | SM | 0.49 | 0.10 | 0.39 |
| 41 | SM | -6.05 | -5.85 | -0.20 |  | WT | 1.22 | 0.25 | 0.97 |
| 42 | SM | -6.63 | -5.99 | -0.63 |  | WT | 0.80 | 0.69 | 0.11 |
| 43 | SM | -4.61 | -5.95 | 1.34 |  | WT | -0.21 | 0.27 | -0.48 |
| 44 | SM | -4.61 | -6.26 | 1.65 |  | WT | 0.30 | 0.31 | -0.01 |
| 45 | SM | -5.77 | -6.05 | 0.29 |  | WT | -0.01 | 0.18 | -0.20 |
| 46 | SM | -6.07 | -4.49 | -1.59 |  | WT | 0.34 | 0.13 | 0.21 |
| 47 | WT | -5.29 | -5.50 | 0.20 |  | WT | 0.13 | -0.04 | 0.17 |
| 48 | WT | -6.80 | -5.25 | -1.55 |  | WT | -0.08 | 0.22 | -0.29 |
| 49 | WT | -6.67 | -6.29 | -0.38 |  | WT | 0.30 | -0.17 | 0.46 |
| 50 | WT | -5.30 | -5.34 | 0.05 |  | WT | -0.04 | 0.21 | -0.25 |
| 51 | WT | -5.56 | -5.79 | 0.23 |  | WT | 0.74 | 0.26 | 0.48 |
| 52 | WT | -5.87 | -5.51 | -0.36 |  | WT | 0.17 | 0.27 | -0.10 |
| 53 | WT | -6.10 | -5.74 | -0.36 |  | WT | 0.64 | -0.01 | 0.65 |
| 54 | WT | -6.13 | -5.91 | -0.22 |  | WT | 0.74 | 0.08 | 0.66 |
| 55 | WT | -5.66 | -5.26 | -0.40 |  | SM | 0.34 | 0.09 | 0.25 |
| 56 | WT | -4.24 | -4.41 | 0.17 |  | SM | 0.26 | -0.38 | 0.63 |
| 57 | WT | -5.49 | -5.13 | -0.35 |  | WT | 1.07 | 0.23 | 0.84 |
| 58 | WT | -4.89 | -4.97 | 0.09 |  | WT | 0.84 | 0.25 | 0.59 |
| 59 | WT | -6.38 | -5.06 | -1.32 |  | SM | 0.22 | -0.08 | 0.30 |
| 60 | WT | -6.91 | -4.76 | -2.15 |  | WT | 0.12 | 0.09 | 0.03 |
| 61 | WT | -4.87 | -4.80 | -0.07 |  | WT | 0.43 | 0.11 | 0.31 |
| 62 | WT | -5.21 | NA | NA |  | SM | 0.72 | NA | NA |
| 63 | WT | -6.22 | -5.71 | -0.51 |  | WT | 0.50 | -0.23 | 0.73 |
| 64 | WT | -5.96 | -4.56 | -1.40 |  | WT | 0.60 | 0.35 | 0.25 |
| 65 | WT | -5.11 | -5.04 | -0.07 |  | WT | 0.31 | 0.17 | 0.14 |
| 66 | WT | -5.99 | -5.61 | -0.38 |  | WT | 0.97 | 0.19 | 0.78 |
| 67 | WT | -4.40 | -3.89 | -0.51 |  | SM | 0.33 | -0.25 | 0.57 |
| 68 | WT | -6.44 | -5.95 | -0.49 |  | SM | 0.71 | 0.51 | 0.20 |
| 69 | WT | -4.66 | -5.58 | 0.91 |  | SM | 0.78 | 0.21 | 0.56 |
| 70 | WT | -6.73 | -6.55 | -0.18 |  | WT | 0.75 | 0.48 | 0.27 |
| 71 | WT | -4.86 | -5.98 | 1.12 |  | SM | 1.39 | 0.17 | 1.22 |
| 72 | WT | -4.82 | -6.09 | 1.27 |  | MMs | 0.19 | 0.71 | -0.52 |
| 73 | WT | -6.29 | -6.10 | -0.19 |  | WT | 0.83 | 0.28 | 0.56 |
| 74 | WT | -5.34 | -5.81 | 0.48 |  | WT | 0.03 | 0.36 | -0.33 |
| 75 | WT | -5.79 | -4.91 | -0.88 |  | WT | 0.71 | 0.35 | 0.36 |
| 76 | WT | -5.48 | -4.21 | -1.28 |  | WT | 0.02 | 0.19 | -0.17 |
| 77 | WT | -5.25 | -5.24 | -0.02 |  | WT | 0.78 | 0.07 | 0.71 |
| 78 | WT | -5.11 | -6.48 | 1.37 |  | WT | 0.67 | 0.37 | 0.31 |
| 79 | WT | -6.41 | -5.16 | -1.25 |  | MMs | 0.87 | -0.19 | 1.06 |
| 80 | WT | -5.35 | -6.63 | 1.28 |  | WT | 0.19 | 0.05 | 0.13 |
| 81 | WT | -6.27 | -4.57 | -1.70 |  | WT | 0.27 | 0.15 | 0.12 |
| 82 | WT | -6.32 | -5.59 | -0.72 |  | WT | 0.58 | 0.03 | 0.55 |
| 83 | WT | -5.18 | -3.02 | -2.16 |  | WT | 0.29 | 0.11 | 0.17 |
| 84 | WT | -6.30 | -4.84 | -1.46 |  | SM | -0.04 | -0.24 | 0.21 |
| 85 | WT | -4.85 | -5.83 | 0.98 |  | WT | 0.22 | -0.43 | 0.64 |
| 86 | WT | -4.90 | -4.87 | -0.03 |  | WT | 0.47 | 0.13 | 0.34 |
| 87 | WT | -5.61 | -5.98 | 0.37 |  | WT | -0.01 | -0.37 | 0.35 |
| 88 | WT | -5.94 | -2.54 | -3.40 |  | WT | 0.28 | -0.38 | 0.67 |
| 89 | WT | -5.69 | -3.77 | -1.92 |  | WT | 0.51 | 0.31 | 0.20 |
| 90 | WT | -4.65 | -5.44 | 0.80 |  | SM | 0.85 | 0.03 | 0.82 |
| 91 | WT | -5.19 | -4.81 | -0.38 |  | SM | 0.42 | 0.05 | 0.37 |
| 92 | WT | -5.67 | -4.06 | -1.61 |  | WT | 0.28 | 0.06 | 0.22 |
| 93 | WT | -5.33 | -6.50 | 1.17 |  | SM | 0.72 | 0.27 | 0.45 |
| 94 | WT | -5.35 | -5.74 | 0.39 |  | WT | 1.07 | -0.16 | 1.23 |
| 95 | WT | -6.23 | -6.03 | -0.20 |  | WT | 0.36 | -0.65 | 1.01 |
| 96 | WT | -5.92 | -5.31 | -0.61 |  | SM | 0.80 | -0.48 | 1.28 |
| 97 | WT | -6.72 | -6.46 | -0.26 |  | WT | 0.71 | 0.26 | 0.44 |
| 98 | WT | -7.13 | -6.27 | -0.85 |  | WT | 1.18 | 0.64 | 0.54 |
| 99 | WT | -6.24 | -6.41 | 0.17 |  | WT | 0.11 | 0.05 | 0.06 |
| 100 | WT | -6.72 | -6.34 | -0.38 |  | SM | 1.15 | 0.23 | 0.92 |
| 101 | WT | -7.13 | -6.49 | -0.63 |  | WT | 0.30 | 0.07 | 0.24 |
| 102 | WT | -3.57 | -6.36 | 2.79 |  | WT | 0.40 | 0.06 | 0.34 |
| 103 | WT | -6.66 | -5.37 | -1.28 |  | WT | 0.34 | 0.02 | 0.32 |
| 104 | WT | -6.20 | -6.46 | 0.26 |  | SM | 0.39 | 0.44 | -0.05 |
| 105 | WT | -5.57 | -5.75 | 0.18 |  | MMs | 2.54 | 0.11 | 2.43 |
| 106 | WT | -4.64 | -4.68 | 0.04 |  | SM | -0.01 | 0.00 | -0.01 |
| 107 | WT | -4.55 | -6.45 | 1.90 |  | WT | 1.67 | 0.42 | 1.25 |
| 108 | WT | -5.78 | -4.39 | -1.40 |  | WT | 0.25 | -0.14 | 0.38 |
| 109 | WT | -6.60 | -6.15 | -0.44 |  | SM | 0.35 | 0.34 | 0.01 |
| 110 | WT | -6.06 | -5.43 | -0.63 |  | SM | 1.68 | 0.51 | 1.17 |
| 111 | WT | -5.43 | -6.12 | 0.69 |  | WT | 0.56 | 0.29 | 0.27 |
| 112 | WT | -5.39 | -6.59 | 1.20 |  | SM | 0.67 | -0.02 | 0.70 |
| 113 | WT | -6.01 | -5.78 | -0.24 |  | WT | 0.79 | 0.52 | 0.27 |
| 114 | WT | -6.76 | -5.66 | -1.10 |  | WT | 0.21 | 0.11 | 0.10 |
| 115 | WT | -5.88 | -5.57 | -0.31 |  | MMs | 0.77 | 0.57 | 0.19 |
| 116 | WT | -6.04 | -5.91 | -0.13 |  | SM | 0.70 | 0.03 | 0.66 |
| 117 | WT | -6.09 | -5.85 | -0.25 |  | WT | 0.46 | 0.26 | 0.20 |
| 118 | WT | -6.28 | -5.97 | -0.31 |  | WT | 0.95 | 0.31 | 0.65 |
| 119 | WT | -6.43 | -5.93 | -0.50 |  | WT | 1.29 | 0.23 | 1.06 |
| 120 | WT | -6.96 | -5.15 | -1.81 |  | WT | 0.60 | 0.47 | 0.13 |
| 121 | WT | -6.45 | -6.38 | -0.07 |  | WT | 1.36 | 0.41 | 0.95 |
| 122 | WT | -6.67 | -6.32 | -0.35 |  | WT | 0.09 | 0.31 | -0.22 |
| 123 | WT | -6.19 | -6.30 | 0.11 |  | WT | 0.53 | 0.05 | 0.48 |
| 124 | WT | -6.16 | -6.27 | 0.11 |  | WT | 0.93 | 0.12 | 0.81 |
| 125 | WT | -7.06 | -6.71 | -0.35 |  | WT | 0.23 | 0.19 | 0.03 |
| 126 | WT | -6.80 | -6.39 | -0.42 |  | WT | 1.31 | 0.32 | 1.00 |
| 127 | WT | -6.32 | -6.29 | -0.03 |  | WT | -0.07 | 0.23 | -0.30 |
| 128 | WT | -6.22 | -3.50 | -2.72 |  | SM | 0.25 | 0.17 | 0.08 |
| 129 | WT | -7.44 | -6.51 | -0.93 |  | WT | 0.90 | -0.02 | 0.92 |
| 130 | WT | -5.47 | -5.60 | 0.13 |  | WT | 0.42 | -0.15 | 0.57 |
| 131 | WT | -5.40 | -5.41 | 0.01 |  | WT | 0.29 | 0.41 | -0.12 |
| 132 | WT | -6.04 | -5.71 | -0.33 |  | MMs | 0.40 | -0.64 | 1.04 |
| 133 | WT | -5.38 | -5.63 | 0.24 |  | WT | 0.24 | -0.02 | 0.26 |
| 134 | WT | -6.64 | -5.72 | -0.91 |  | WT | 0.72 | -0.39 | 1.12 |
| 135 | WT | -5.82 | -5.58 | -0.23 |  | SM | 1.30 | 0.42 | 0.87 |
| 136 | WT | -6.03 | -5.65 | -0.38 |  | SM | 1.36 | 0.78 | 0.58 |
| 137 | WT | -7.11 | -6.54 | -0.56 |  | SM | 1.14 | 0.55 | 0.59 |
| 138 | WT | -6.13 | -5.08 | -1.05 |  | SM | 1.78 | 0.39 | 1.39 |
| 139 | WT | -5.38 | -4.42 | -0.96 |  | WT | 0.92 | 0.52 | 0.41 |
| 140 | WT | -6.42 | -5.90 | -0.52 |  | SM | 0.28 | -0.09 | 0.36 |
| 141 | WT | -5.53 | -5.77 | 0.24 |  | SM | -0.02 | 0.18 | -0.20 |
| 142 | WT | -5.64 | -6.19 | 0.56 |  | WT | 1.66 | 0.30 | 1.35 |
| 143 | WT | -5.98 | -5.22 | -0.76 |  | WT | 0.06 | -0.12 | 0.18 |
| 144 | WT | -6.79 | -4.95 | -1.84 |  | WT | 0.19 | -0.25 | 0.44 |
| 145 | WT | -5.80 | -5.16 | -0.64 |  | WT | 0.78 | -0.38 | 1.16 |
| 146 | WT | -4.91 | -5.91 | 1.00 |  | SM | 1.61 | 1.16 | 0.46 |
| 147 | WT | -6.50 | -5.75 | -0.76 |  | SM | 1.69 | -0.02 | 1.71 |
| 148 | WT | -6.71 | -6.01 | -0.70 |  | SM | 2.02 | 0.36 | 1.66 |
| 149 | WT | -6.50 | -5.95 | -0.55 |  | WT | 0.20 | 0.00 | 0.20 |
| 150 | WT | -6.52 | -6.12 | -0.40 |  | WT | 0.49 | -0.14 | 0.63 |
| 151 | WT | -6.13 | -5.63 | -0.50 |  | WT | 0.49 | 0.20 | 0.29 |
| 152 | WT | -6.27 | -5.98 | -0.29 |  | WT | 0.80 | 0.50 | 0.30 |
| 153 | WT | -5.94 | -5.61 | -0.33 |  | SM | 1.23 | -0.45 | 1.69 |
| 154 | WT | -5.74 | -5.56 | -0.18 |  | WT | 0.50 | 0.34 | 0.16 |
| 155 | WT | -6.69 | -6.09 | -0.60 |  | WT | 1.06 | 0.01 | 1.05 |
| 156 | WT | -5.08 | -5.70 | 0.62 |  | WT | 1.47 | -0.46 | 1.92 |
| 157 | WT | -5.06 | -4.73 | -0.33 |  | SM | 0.92 | 0.30 | 0.62 |
| 158 | WT | -6.13 | -3.84 | -2.29 |  | WT | 0.93 | 0.04 | 0.89 |
| 159 | WT | -5.68 | -5.54 | -0.14 |  | SM | 0.85 | 0.05 | 0.80 |
| 160 | WT | -6.26 | -5.98 | -0.28 |  | SM | 1.57 | 0.54 | 1.03 |
| 161 | WT | -5.91 | -5.27 | -0.64 |  | SM | 0.45 | -0.36 | 0.81 |
| 162 | WT | -6.29 | -6.10 | -0.19 |  | WT | 0.51 | 0.33 | 0.18 |
| 163 | WT | -6.27 | -6.29 | 0.03 |  | SM | 0.51 | -0.07 | 0.57 |
| 164 | WT | -4.99 | -5.76 | 0.76 |  | SM | -0.10 | -0.67 | 0.57 |
| 165 | WT | -6.20 | -6.47 | 0.27 |  | SM | 1.04 | 0.16 | 0.88 |
| 166 | WT | -6.38 | -6.10 | -0.28 |  | WT | 0.40 | 0.15 | 0.25 |
| 167 | WT | -6.02 | -6.64 | 0.62 |  | WT | 0.57 | 0.31 | 0.26 |
| 168 | WT | NA | -6.05 | NA |  | SM | NA | 0.48 | NA |
| 169 | WT | -6.25 | -6.89 | 0.63 |  | SM | 1.27 | -0.27 | 1.54 |
| 170 | WT | -6.87 | -6.36 | -0.51 |  | SM | 2.16 | 0.08 | 2.08 |
| 171 | WT | -6.59 | -6.47 | -0.12 |  | WT | 0.73 | -0.44 | 1.17 |
| 172 | WT | -6.54 | -5.29 | -1.25 |  | WT | 0.77 | 0.05 | 0.72 |
| 173 | WT | -7.30 | -6.60 | -0.70 |  | WT | 0.70 | 0.07 | 0.63 |
| 174 | WT | -5.63 | -6.59 | 0.96 |  | WT | 0.44 | 0.05 | 0.39 |
| 175 | WT | -4.95 | -4.86 | -0.09 |  | WT | 1.41 | 0.55 | 0.87 |
| 176 | WT | -5.73 | -5.56 | -0.16 |  | SM | 0.46 | 0.55 | -0.09 |
| 177 | WT | NA | -6.18 | NA |  | WT | NA | 0.23 | NA |
| 178 | WT | -6.44 | -6.06 | -0.38 |  | SM | 0.91 | 0.68 | 0.22 |
| 179 | WT | -7.10 | -6.11 | -0.99 |  | WT | 0.50 | 0.07 | 0.43 |
| 180 | WT | -6.20 | -5.68 | -0.52 |  | WT | 0.83 | 0.38 | 0.45 |
| 181 | WT | -6.09 | -5.84 | -0.25 |  | WT | 0.83 | -0.29 | 1.12 |
| 182 | WT | -5.87 | -5.60 | -0.28 |  | WT | 0.58 | 0.35 | 0.23 |
| 183 | WT | -6.73 | -5.72 | -1.01 |  | WT | 0.22 | 0.19 | 0.03 |
| 184 | WT | -6.37 | -4.65 | -1.72 |  | WT | 0.96 | 0.34 | 0.62 |
| 185 | WT | -5.04 | -5.67 | 0.63 |  | SM | 0.77 | -0.46 | 1.23 |
| 186 | WT | -4.56 | -6.14 | 1.58 |  | SM | 0.54 | 0.18 | 0.36 |
| 187 | WT | -4.55 | -6.14 | 1.59 |  | WT | 1.01 | 0.43 | 0.58 |
| 188 | WT | -5.34 | -5.14 | -0.20 |  | WT | 0.68 | 0.37 | 0.30 |
| 189 | WT | -6.72 | -6.47 | -0.25 |  | WT | 1.05 | -0.04 | 1.09 |
| 190 | WT | -4.66 | -5.89 | 1.23 |  | WT | 0.28 | -0.32 | 0.60 |
| 191 | WT | -5.65 | -6.39 | 0.74 |  | WT | 1.09 | 0.12 | 0.97 |
| 192 | WT | -7.02 | -6.48 | -0.55 |  | WT | 1.32 | 0.45 | 0.87 |
| 193 | WT | -6.45 | -5.86 | -0.59 |  | WT | 0.76 | 0.23 | 0.53 |
| 194 | WT | -5.18 | -5.37 | 0.19 |  | SM | 0.74 | 0.14 | 0.60 |
| 195 | WT | -6.42 | -5.61 | -0.81 |  | WT | 0.19 | 0.01 | 0.18 |
| 196 | WT | -4.83 | -6.22 | 1.39 |  | WT | 0.19 | -0.02 | 0.20 |
| 197 | WT | -5.65 | -6.08 | 0.43 |  | WT | 0.51 | 0.41 | 0.10 |
| 198 | WT | -6.37 | -6.44 | 0.07 |  | WT | 0.72 | 0.37 | 0.35 |
| 199 | WT | -7.08 | -5.01 | -2.08 |  | WT | 0.53 | -0.09 | 0.62 |
| 200 | WT | -5.53 | -5.77 | 0.24 |  | SM | 0.86 | -0.01 | 0.87 |
| 201 | WT | -6.43 | -5.82 | -0.61 |  | WT | 0.20 | -0.21 | 0.41 |
| 202 | WT | -5.81 | -5.85 | 0.05 |  | WT | 0.30 | -0.05 | 0.35 |
| 203 | WT | -5.62 | NA | NA |  | SM | 0.85 | NA | NA |
| 204 | WT | -5.14 | -4.95 | -0.20 |  | WT | 0.74 | -0.02 | 0.76 |
| 205 | WT | -6.86 | -5.60 | -1.26 |  | WT | 0.51 | 0.17 | 0.34 |
| 206 | WT | -6.54 | -5.81 | -0.73 |  | SM | 0.28 | 0.00 | 0.28 |
| 207 | WT | -5.59 | -5.53 | -0.06 |  | SM | 0.23 | 0.10 | 0.13 |
| 208 | WT | -4.93 | -5.32 | 0.39 |  | WT | 0.49 | 0.16 | 0.33 |
| 209 | WT | -6.12 | -4.72 | -1.40 |  | SM | 0.97 | -0.40 | 1.38 |
| 210 | WT | -4.89 | -5.61 | 0.72 |  | WT | 0.90 | 0.35 | 0.55 |
| 211 | WT | -5.32 | -5.45 | 0.13 |  | WT | 0.23 | 0.24 | -0.01 |
| 212 | WT | -5.90 | -5.52 | -0.39 |  | SM | 0.61 | -0.09 | 0.70 |
| 213 | WT | -5.29 | -6.03 | 0.74 |  | WT | 1.04 | 0.19 | 0.84 |
| 214 | WT | -6.11 | -5.53 | -0.59 |  | WT | 0.66 | -0.23 | 0.88 |
| 215 | WT | -5.18 | -5.77 | 0.60 |  | WT | 0.37 | -0.18 | 0.55 |
| 216 | WT | -4.75 | -5.29 | 0.54 |  | WT | 0.31 | -0.55 | 0.86 |
| 217 | WT | -5.96 | -5.77 | -0.19 |  | WT | 0.29 | 0.23 | 0.05 |
| 218 | WT | -7.03 | -4.72 | -2.31 |  | WT | 0.74 | 0.33 | 0.41 |
| 219 | WT | -6.36 | -4.81 | -1.55 |  | SM | 1.66 | -0.18 | 1.84 |
| 220 | WT | -6.80 | -5.62 | -1.18 |  | WT | 0.06 | 0.41 | -0.35 |
| 221 | WT | -6.80 | -6.37 | -0.43 |  | SM | 0.58 | -0.08 | 0.65 |
| 222 | WT | NA | NA | NA |  | WT | NA | NA | NA |
| 223 | WT | NA | NA | NA |  | WT | NA | NA | NA |
